# Defining the Benefits of Antibiotic Resistance in Commensals and the Scope for Resistance Optimization

**DOI:** 10.1128/mbio.01349-22

**Published:** 2022-12-07

**Authors:** Kristofer Wollein Waldetoft, Sarah Sundius, Rachel Kuske, Sam P. Brown

**Affiliations:** a School of Biological Sciences, Georgia Institute of Technology, Atlanta, Georgia, USA; b Center for Microbial Dynamics and Infection, Georgia Institute of Technology, Atlanta, Georgia, USA; c Torsby Hospital, Torsby, Sweden; d Interdisciplinary Program in Quantitative Biosciences, Georgia Institute of Technology, Atlanta, Georgia, USA; e School of Mathematics, Georgia Institute of Technology, Atlanta, Georgia, USA; Emory University

**Keywords:** antibiotic resistance, bystander selection, commensals, competitive release, ecological interactions, mathematical modeling, microbial dynamics, microbial ecology, microbiome, polymicrobial infection

## Abstract

Antibiotic resistance is a major medical and public health challenge, characterized by global increases in the prevalence of resistant strains. The conventional view is that all antibiotic resistance is problematic, even when not in pathogens. Resistance in commensal bacteria poses risks, as resistant organisms can provide a reservoir of resistance genes that can be horizontally transferred to pathogens or may themselves cause opportunistic infections in the future. While these risks are real, we propose that commensal resistance can also generate benefits during antibiotic treatment of human infection, by promoting continued ecological suppression of pathogens. To define and illustrate this alternative conceptual perspective, we use a two-species mathematical model to identify the necessary and sufficient ecological conditions for beneficial resistance. We show that the benefits are limited to species (or strain) interactions where commensals suppress pathogen growth and are maximized when commensals compete with, rather than prey on or otherwise exploit pathogens. By identifying benefits of commensal resistance, we propose that rather than strictly minimizing all resistance, resistance management may be better viewed as an optimization problem. We discuss implications in two applied contexts: bystander (nontarget) selection within commensal microbiomes and pathogen treatment given polymicrobial infections.

## INTRODUCTION

Antimicrobial resistance is normally viewed as categorically dangerous, regardless of where it is found (1–3). Here, we develop an opposing view, that antimicrobial resistance in commensals can support improved antibiotic treatment outcomes with respect to both target and nontarget pathogens, dependent on the details of resistance mechanisms and microbiome community interaction structure.

The ensemble of microbes that live intimately with us are collectively termed the human microbiome (used here inclusively of organisms and their genomes [4]) and can play a key role in host defense against pathogen invasion (5). While antibiotic resistance is conventionally tracked in pathogen species only, an increasing number of studies point to a rising prevalence of antimicrobial resistance in nonpathogen species (commensals) that are found in our microbiomes (6–9). These increases are unsurprising given the frequent exposure of commensal organisms to antibiotics. Here, we ask, can commensal resistance ever be beneficial?

The starting point for this investigation is a perceived tension between two widely held views concerning commensal organisms. First, antibiotic resistance in commensal bacteria is considered problematic for resistance management (7). The reasons are that commensals can horizontally transfer their resistance genes to pathogens (9–11), and in addition, they may themselves cause opportunistic infections (12). Second, commensals are thought to protect the host from pathogens (13, 14), providing colonization resistance (15–17), competitive suppression (18), and even suppression of pathogen evolution (19–21), but antibiotic treatment disrupts these ecological functions of commensals (22–24)—unless they are sufficiently resistant. Hence the tension. The presence of resistance in commensals is assumed to be negative for resistance management and public health, but there is a logical argument that under antibiotic treatment, resistant commensals can continue to protect against uncontrolled growth of target or nontarget pathogens within patients, under levels of antibiotic exposure that would remove antibiotic-sensitive commensals. In support of this argument, experimental studies have demonstrated that antibiotic exposure in drug-susceptible communities can lead to an increase in the absolute density of a target pathogen—a process known as “competitive release” (22–25).

A key assumption of our argument is that pathogens interact (competitively or otherwise) with commensal organisms during the course of infection treatment. This assumption defines the applied scope of the work, which is limited to the context of (i) chronic polymicrobial infections and (ii) bystander selection. Chronic (long-term) infections commonly consist of multispecies communities (26) and can increase morbidity and mortality in affected individuals, posing a rising burden on health care systems due to increases in at-risk populations (27, 28). In the case of people with cystic fibrosis, polymicrobial lung infections can persist for decades despite intensive exposure to antibiotics (26). A striking meta-analysis of target pathogen drug susceptibility testing in people with cystic fibrosis showed that antibiotic susceptibility test results had no correlation with patient health outcomes following treatment (29)—raising the potential that the distribution of resistances across multiple organisms may be confounding the relationship between antibiotic exposure and treatment outcomes.

In contrast to chronic infections, acute (rapidly resolved) infections tend to display low diversity and thus are not the direct focus of this study. Yet treatment of acute infections can also trigger questions of commensal resistance and competitive release, due to the incidental exposure of other microbiome sites to antibiotics (30). Tedijanto et al. defined bystander selection as the selective pressure exerted by antibiotics on microbes that are not the target pathogen of treatment and documented that, for many pathogens, bystander selection is approximately 10-fold more common than selection imposed on pathogens when they are the target of treatment (30). The dominance of bystander selection reflects the opportunistic lifestyle of many bacterial pathogens (12), where pathogens can spend much of their time living harmlessly within commensal microbiome sites, such as the gut or nasopharynx. While harmless at one point in time, carriage of opportunistic pathogens is a risk factor for subsequent infection, and the risk is magnified if the pathogen is drug resistant (31). Bystander exposures can have large impacts on microbiome composition and can trigger competitive release of previously rare (nontarget) pathogens at both the infection site (25) and other locations (32, 33), for instance, contributing to dangerous Clostridium difficile infections (17, 34, 35).

**FIG 1:**
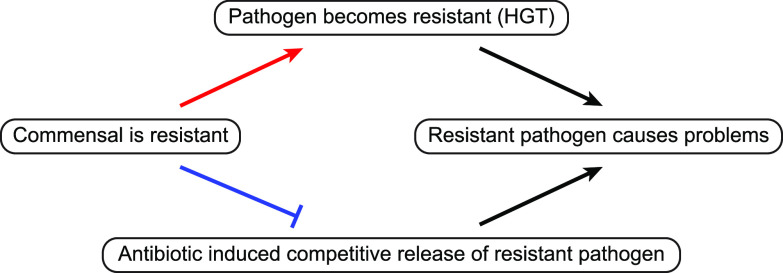
Potential costs and benefits of antibiotic resistance in commensal organisms. From a medical perspective, antibiotic resistance in a commensal may have both positive and negative effects. For example, a resistant commensal may transfer a resistance gene horizontally (HGT) to a pathogen, but it may also limit competitive release of a resistant pathogen under antibiotic exposure. The net effect of commensal resistance on the problem of resistance in pathogens is therefore nonobvious. Arrows denote causation; the barred arrow denotes prevention.

Our argument that resistance can confer benefits as well as costs coincides with a recent brief commentary on antibiotic resistance in the gut, where Gautam Dantas stated that “an enriched gut resistome is a double-edged sword” (36). This highlights that considering the benefits of resistance is a complex challenge, which will require careful balance of the risks of resistance acquisition and the benefits of ecological suppression (36). Taking a mathematical modeling approach allows us to provide a proof of concept (37), identifying the combinations of ecological factors that can generate beneficial resistance. To explore the logic and limits of our proposal of beneficial resistance, we develop and analyze a two-species mathematical model, allowing us to precisely identify the conditions where commensal resistance can provide treatment benefits. Figure 1 sketches the structure of the reasoning underlying the study; the blue barred arrow represents the aspect on which we focus.

## RESULTS

**FIG 2:**
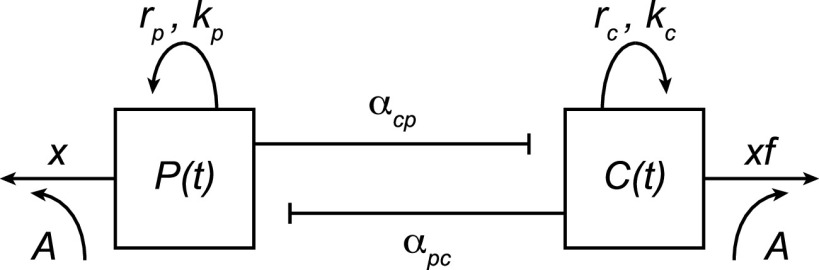
Mathematical model schematic. The population dynamics of a pathogen population [absolute density *P*(*t*)] and a commensal population [absolute density *C*(*t*)] are governed by an extension of the two-species competitive Lotka-Volterra model, specifically, the coupled ordinary differential equations (equation 3 in the text; see Materials and Methods).

Our primary mathematical analysis focuses on mapping the potential benefits of commensal resistance (the blue arrow in Fig. 1, limiting pathogen expansion under drug exposure). Specifically, we build and analyze a two-species (or strain) community ecological model and examine the impact of various drug exposures, commensal resistance, and the ecological interactions between the two species. Our baseline approach uses a modified Lotka-Volterra competition model, describing the population dynamics of a pathogen species (absolute density *P*) and a commensal species (absolute density *C*), subject to antibiotic perturbation *A* (Fig. 2, Materials and Methods). We focus on a Lotka-Volterra model, as it allows for a range of ecological relationships (competition, exploitation, mutualism) in a single and tractable mathematical framework and is now the dominant framework in microbiome modeling studies (35, 38, 39). To assess the robustness of our Lotka-Volterra analyses, we also conduct numerical explorations of more complex models (introducing explicit resources and spatial dynamics) to demonstrate that our qualitative results are not bound to a specific model framework.

Note that while we use the language of community ecological species interactions, our model also serves to capture interactions between conspecific strains, for example, a commensal versus a pathogenic strain of Escherichia coli. In line with our two applied contexts, the antibiotic perturbation may be triggered by the focal pathogen *P* (commonly, but not necessarily, the case in polymicrobial infection treatment) or by another pathogen in the community or at another body site (bystander selection).

Each species has its own maximal growth rate (*r_p_*, *r_c_*) and single-species carrying capacity (*k_p_*, *k_c_*). Species interaction coefficients, *α_ij_*, capture the per-capita inhibitory impact of species *j* on species *i* (note that we allow for negative signed interaction coefficients, therefore allowing mutualism [both coefficients negative], exploitation [one positive, one negative], and competition [both positive]). The antibiotic terms follow standard approaches (34, 39, 40) by capturing antibiotic inhibition (whether bactericidal or bacteriostatic) via an additional density-independent loss term. Antibiotic perturbation, 0 < *A* ≤ 1, scales the pathogen loss rate from 0 to its maximal clearance rate, *x* (equation 3a, below). In order to address commensal resistance, we introduce the relative susceptibility parameter, *f*, which modifies *x*, capturing the extent to which the commensal is more (*f* > 1) or less (*f* < 1) susceptible to antibiotic clearance than the pathogen (same resistance when *f* = 1) (equation 3b, below). Table S1 in the supplemental material provides a summary of parameter definitions.

10.1128/mbio.01349-22.2 TABLE S1 Definition of model parameters and summary of specific parameter values and ranges used to generate figures. Download Table S1, DOCX file, 0.01 MB. Copyright © 2022 Wollein Waldetoft et al. 2022 Wollein Waldetoft et al. https://creativecommons.org/licenses/by/4.0/ This content is distributed under the terms of the Creative Commons Attribution 4.0 International license.

A stability analysis of the model in Fig. 2 (equation 3) illustrates that this system of equations can lead to diverse locally stable outcomes, including pathogen dominance (P* > 0, C* = 0), commensal dominance (P* = 0, C* > 0), coexistence (P* > 0, C* > 0), or joint extinction (P* = C* = 0) (see Text S1 and Table S2 for a brief summary). In light of our key assumption that the commensal and pathogen interact during antibiotic exposure, we focus below on scenarios allowing coexistence in the absence of antibiotic treatment (i.e., chronic infections, bystander microbiomes, and non-acute clonal infections). In these polymicrobial scenarios, prior to antibiotic exposures, both organisms are present and are at or will tend toward an ecologically stable coexistence equilibrium. We then ask, what is the impact of relative commensal susceptibility *f* as a function of antibiotic exposure *A* and other ecological parameters (growth rates, carrying capacities, interaction signs, and magnitudes)? We focus both on qualitative outcomes—the transition from coexistence to pathogen dominance, where the commensal population is eliminated—and quantitative outcomes—the net change in density of the pathogen following antibiotic exposure. In particular, our general mathematical framework allows us to define precise conditions of “competitive release” (where pathogen density increases with antibiotic exposure [22–25]) and “beneficial resistance” (where pathogen density decreases with commensal resistance). We also discuss what these results mean in terms of previously rare or invading pathogens.

10.1128/mbio.01349-22.1 TEXT S1 Supplemental Information. Text detailing mathematical analyses, including the discussion of results given variation of growth and ecological parameter values, model architecture, and the addition of established costs of commensal resistance (Fig. S1 to S6). Download Text S1, PDF file, 0.3 MB. Copyright © 2022 Wollein Waldetoft et al. 2022 Wollein Waldetoft et al. https://creativecommons.org/licenses/by/4.0/ This content is distributed under the terms of the Creative Commons Attribution 4.0 International license.

10.1128/mbio.01349-22.3 TABLE S2 Ecological outcomes and stability conditions given antibiotic exposure (equation 3). Download Table S2, DOCX file, 0.01 MB. Copyright © 2022 Wollein Waldetoft et al. 2022 Wollein Waldetoft et al. https://creativecommons.org/licenses/by/4.0/ This content is distributed under the terms of the Creative Commons Attribution 4.0 International license.

### Resource competition.

**FIG 3:**
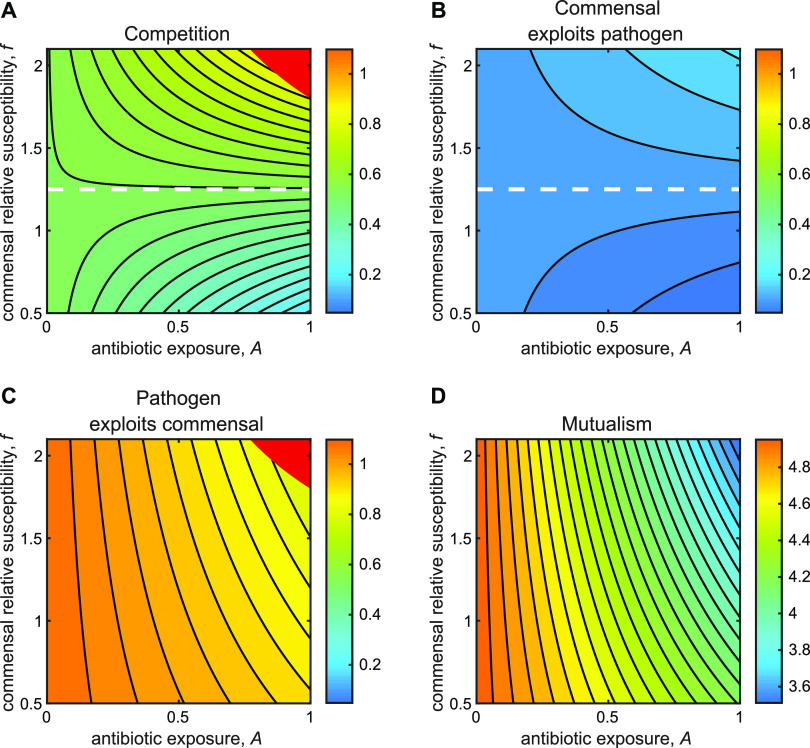
Commensal resistance can enhance pathogen clearance, depending on ecological interactions. Contour plots are of pathogen density at coexistence equilibrium (P*, C*), $P^{*}=\frac{r_{c}k_{p}\left(r_{p}\;-\;\mathrm{xA}\right)\;-\;\alpha_{\mathrm{pc}}r_{p}k_{c}\left(r_{c}\;-\;\mathrm{xfA}\right)}{r_{p}r_{c}\left(1\;-\;\alpha_{\mathrm{pc}}\alpha_{\mathrm{cp}}\right)}$ as a function of antibiotic concentration *A* (*x* axis) and relative commensal susceptibility *f* (*y* axis) for four distinct ecological interactions between pathogen and commensal. (A) Competitive interaction (*α_pc_* > 0, *α_cp_* > 0). Competitive release occurs at higher values of commensal susceptibility *f* (above the dashed white line, $f^{*}=\frac{r_{c}k_{p}}{r_{p}k_{c}\alpha_{\mathrm{pc}}}$) (B) Commensal exploitation of pathogen (*α_pc_* > 0, *α_cp_* < 0). (C) Pathogen exploitation of commensal (*α_pc_* < 0, *α_cp_* > 0). (D) Mutualism (*α_pc_* < 0, *α_cp_* < 0). Note that the color bars are consistent between panels A to C with standardized contour increments of 0.03. Panel D uses a different color scale and a contour increment of 0.06 due to large magnitude differences compared to the other scenarios. Red regions (panels A and C) indicate that coexistence is unstable and pathogen dominance is stable. Specific parameter values: *r_p_* = *r_c_* = 0.5, *k_p_* = *k_c_* = 1, *x* = 0.1 for all (panels A to D); (A) *α_pc_* = 0.8, *α_cp_* = 0.8; (B) *α_pc_* = 0.8, *α_cp_* = −0.8; (C) *α_pc_* = −0.8, *α_cp_* = 0.8; (D) *α_pc_* = −0.8, *α_cp_* = −0.8. In Text S1 and Fig. S1 to S4, we explore variations of these parameters to support the generalization of our results.

In our first analysis, we consider the case where the two species compete for limiting resources, implying *α_pc_* > 0 and *α_cp_* > 0. In Fig. 3A, we plot the equilibrium pathogen density, P*, following antibiotic exposure, as a function of antibiotic perturbation, *A*, and the relative susceptibility of the commensal *f.* Figure 3A illustrates that when commensals are relatively susceptible to the antibiotic (high *f*; above the white dashed line), we recover the classic competitive release scenario: higher antibiotic exposure *A* triggers a net expansion of pathogen density P*. In contrast, when commensals are relatively resistant (low *f*), we see a net decrease in pathogen load with increased antibiotic (exceeding the impact of antibiotic alone). In other words, commensal resistance can reduce total pathogen abundance, due to a combination of antibiotic killing and competitive suppression. In a target treatment context (polymicrobial infection), commensal resistance will enhance the efficacy of treatment by reducing the target pathogen load. In a nontarget bystander context, commensal resistance will block the expansion of a previously rare or opportunistic pathogen, potentially even preventing colonization (Text S1, Fig. S1 and S2).

10.1128/mbio.01349-22.5 FIG S1 The commensal has a higher growth rate than the pathogen, *r_c_* > *r_p_*. Contour maps of pathogen equilibrium density, P*, at stable coexistence (equation S7). Dark blue regions in panels A and B indicate where coexistence is unstable and commensal dominance (equation S3) is stable. Competitive release will not occur in any interaction scenario for the chosen ranges of *f* and *A*. Color scales are standardized for panels A to C with contour increments of 0.03. Panel D has its own color scale and contour increments of 0.06 to account for larger densities. Specific parameters: *r_p_* = 0.25, *r_c_* = 0.75, *k_p_* = 1, *k_c_* = 1, and *x* = 0.1. Interaction coefficients (*α_pc_*, *α_cp_*) for panels (A to D) are consistent with Fig. 3 and Table S3. Download FIG S1, EPS file, 1.8 MB. Copyright © 2022 Wollein Waldetoft et al. 2022 Wollein Waldetoft et al. https://creativecommons.org/licenses/by/4.0/ This content is distributed under the terms of the Creative Commons Attribution 4.0 International license.

10.1128/mbio.01349-22.4 TABLE S3 Summary of four types of possible ecological interaction scenarios. Download Table S3, DOCX file, 0.01 MB. Copyright © 2022 Wollein Waldetoft et al. 2022 Wollein Waldetoft et al. https://creativecommons.org/licenses/by/4.0/ This content is distributed under the terms of the Creative Commons Attribution 4.0 International license.

10.1128/mbio.01349-22.6 FIG S2 The commensal has higher carrying capacity than the pathogen, *k_c_* > *k_p_*. Contour maps of pathogen equilibrium density, P*, at stable coexistence (equation S7). Dark blue regions in panels A and B indicate that coexistence is unstable for all *f* and *A*, and commensal dominance (equation S3) is stable. Competitive release will not occur in any interaction scenario for the chosen ranges of *f* and *A*. Color scales are standardized for panels A to C with contour increments of 0.03. Panel D has its own color scale and contour increments of 0.06 to account for larger densities. Specific parameters: *r_p_* = 0.5 *r_c_* = 0.5, *k_p_* = 0.5, *k_c_* = 1.5, and *x* = 0.1. Interaction coefficients (*α_pc_*, *α_cp_*) for panels A to D are consistent with Fig. 3 and Table S3. Download FIG S2, EPS file, 1.5 MB. Copyright © 2022 Wollein Waldetoft et al. 2022 Wollein Waldetoft et al. https://creativecommons.org/licenses/by/4.0/ This content is distributed under the terms of the Creative Commons Attribution 4.0 International license.

Figure 3A presents a numerical analysis of competition, defined by specific parameter choices on the values of growth, carrying capacity, and interaction coefficients. To explore the generality of the patterns observed in Fig. 3A, we mathematically analyze how pathogen density at coexistence equilibrium P* changes with antibiotic exposure and commensal susceptibility, via inspection of the gradient functions,

$$\frac{\partial P^{*}}{\partial A}=\frac{x\left(\alpha_{\mathrm{pc}}r_{p}k_{c}f\;-\;r_{c}k_{p}\right)}{r_{p}r_{c}\left(1\;-\;\alpha_{\mathrm{pc}}\alpha_{\mathrm{cp}}\right)}\text{ and }\frac{\partial P^{*}}{\partial f}=\frac{\alpha_{\mathrm{pc}}k_{c}xA}{r_{c}\left(1\;-\;\alpha_{\mathrm{pc}}\alpha_{\mathrm{cp}}\right)}$$

(see Materials and Methods and Text S1 for gradient definitions and additional specification of conditions for coexistence, which include the absence of strong interference competition that tends to lead to single-species dominant or bistable outcomes).

The gradient ∂P*/∂*A* (change in equilibrium pathogen density P* with respect to change in antibiotic exposure *A*) provides a precise definition of competitive release given that the commensal competes with the pathogen. Competitive release occurs when increases in antibiotic exposures *A* lead to increases in pathogen density P* (i.e., ∂P*/∂*A* > 0), as a result of removing a competing organism. Using this definition, we find that competitive release will occur (subject to coexistence stability constraints, specifically, we assume |*α_ij_*| < 1; see Materials and Methods and Text S1) if

(1) $$\alpha_{\mathrm{pc}}f>\frac{r_{c}k_{p}}{r_{p}k_{c}}$$

This condition for competitive release captures intuitive effects, namely, that competitive release is more likely when there is greater commensal inhibition of pathogens (larger *α_pc_*) or greater commensal drug susceptibility (higher *f*) (Fig. 3A). In addition, we find that the threshold for competitive release is lowered by higher commensal carrying capacity and lower commensal growth rate (equation 1). If the antibiotic exposure *A* and commensal susceptibility *f* are high enough to transition to the stable pathogen dominance state (red region in Fig. 3A), competitive release is no longer possible, as the commensal population can no longer persist, and the pathogen density will decrease with increasing antibiotic.

The gradient ∂P*/∂*f* (change in pathogen density P* with respect to change in commensal susceptibility *f*) allows a direct examination of the potential benefits of commensal resistance. If commensal resistance is beneficial, then we expect ∂P*/∂*f* > 0 (higher commensal susceptibility leads to greater pathogen burden). From this definition, we find the conditions for beneficial commensal resistance (i.e., ∂P*/∂*f* > 0) to be

(2) $$\frac{\alpha_{\mathrm{pc}}k_{c}xA}{r_{c}\left(1\;-\;\alpha_{\mathrm{pc}}\alpha_{\mathrm{cp}}\right)}>0$$

Given positive growth parameters, this result simplifies to *α_pc_* > 0, implying that increased commensal resistance is always beneficial under antibiotic treatment (*A* > 0), given pathogen-commensal competition, or more generally, when the commensal inhibits the pathogen—because commensal resistance mitigates competitive release. We also find that the marginal benefit of increasing commensal resistance is positively dependent on the maximal degree of antibiotic clearance of the pathogen, *x* (equation 2), or in other words, how resistant the pathogen is.

The results discussed above and shown in Fig. 3A assume competitive community interactions. While experimental assessment of microbial species interactions indicate that competitive interactions are common (e.g., reference 41), they are not ubiquitous, with examples of species exploitation, facilitation, and mutualism frequently observed (41–44). In the following sections, we relax the assumption of competitive interactions by allowing alternate modes of interaction.

### Exploitative and mutualistic interactions.

**FIG 4:**
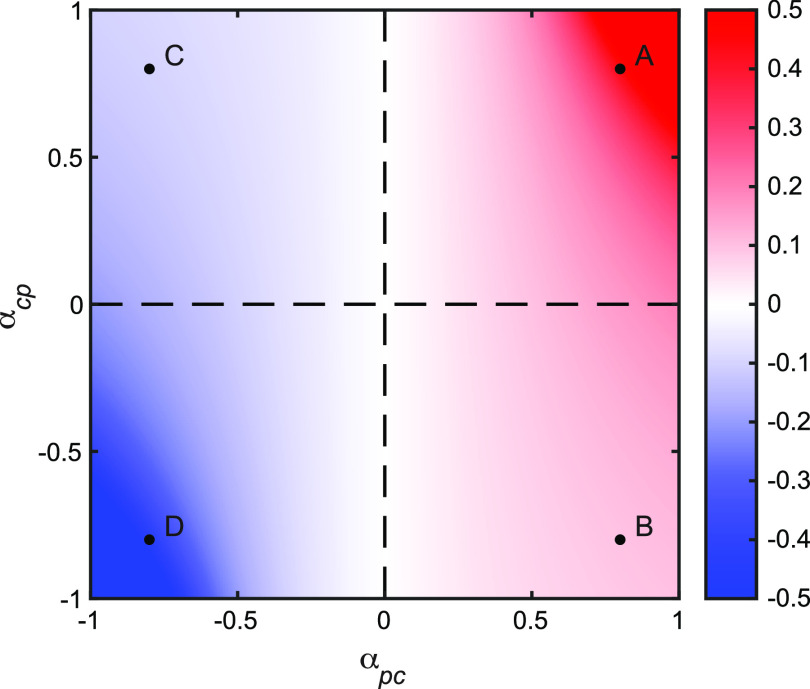
Beneficial resistance (∂P*/∂*f* > 0) is dependent on commensal suppression of the pathogen (*α_pc_* > 0), while magnitude of effect is dependent on reciprocal suppression (*α_pc_* and *α_cp_*). Heat map showing ∂P*/∂*f* given high antibiotic exposure. Dashed lines are defined as *α_pc_* = 0 and *α_cp_* = 0. Positive values of ∂P*/∂*f* (red gradient, region where *α_pc_* > 0) correspond to beneficial commensal resistance, and negative values of ∂P*/∂*f* (blue gradient, region where *α_pc_* < 0) indicate that commensal resistance is not beneficial. Points A to D mark the (*α_pc_*, *α_cp_*) values for the ecological interactions defined in Fig. 3. (A) Competition: (0.8, 0.8), ∂P*/∂*f* = 0.4444; (B) commensal exploits pathogen: (0.8, −0.8), ∂P*/∂*f* = 0.0976; (C) pathogen exploits commensal: (−0.8, 0.8), ∂P*/∂*f* = −0.0976; (D) mutualism: (−0.8, −0.8), ∂P*/∂*f* = −0.4444. Specific parameter values: *r_p_* = *r_c_* = 0.5, *k_p_* = *k_c_* = 1, *x* = 0.1, *A* = 1.

Following our analysis of the competition scenario (Fig. 3A), we introduce the possibility that commensal growth is facilitated by the presence of pathogens (*α_cp_* < 0), for instance, due to predation on the pathogen or cross-feeding on pathogen by-products, while still suppressing pathogen growth (*α_pc_* > 0). In this case (Fig. 3B), we recover an exploitative interaction where commensals gain and pathogens lose from any interaction between the two species. While the parameterization changes (*α_cp_* is now negative), the impacts of changing antibiotic exposure *A* and commensal susceptibility *f* are still governed by equations 1 and 2. From these equations, we see that the same qualitative results apply, as the critical parameter *α_pc_* remains positive. Fig. 4 demonstrates that the quantitative impact of increasing commensal resistance is weaker in the commensal exploitation case (Fig. 3B) than in the competition case (Fig. 3A). A simple intuitive reason for the limited effect (Fig. 3A versus Fig. 3B) is the difference in ecological feedbacks in these two scenarios. In the competitive scenario (Fig. 3A), antibiotic-induced pathogen clearance allows for greater commensal growth, which in turn translates to greater competitive suppression of the pathogen. In contrast, the positive-control feedback of commensal-pathogen competition is not present in the exploitation scenario (Fig. 3B). Here, antibiotic-induced pathogen clearance will now inhibit commensal growth (less pathogen to exploit), which in turn translates to less exploitative suppression of the pathogen (see Text S1 for a mathematical dissection of this effect).

In Fig. 3C, we reverse the direction of exploitation and introduce a scenario where the pathogen is able to exploit the commensal (*α_pc_* < 0, *α_cp_* > 0), for instance, due to pathogen cross-feeding on metabolites produced by commensals. In this case, we see a qualitative shift because *α_pc_* < 0. Applying equation 1 in this scenario, we see that competitive release is no longer possible. Increased antibiotic exposure will always suppress pathogen density (∂P*/∂*A* < 0), as the commensal is now a facilitator of pathogen growth. From equation 2, we see that commensal resistance is never beneficial in limiting pathogen burden in this scenario, highlighting that under antibiotic exposure, pathogen growth is maximized at high commensal resistance.

Finally, we turn to the mutualism scenario, where each species facilitates the growth of the other (Fig. 3D). Applying equations 1 and 2 to this scenario, we see that ∂P*/∂*A* < 0 (there is no competitive release) and ∂P*/∂*f* < 0 (there is no benefit of commensal resistance with respect to pathogen density).

In the mutualism (Fig. 3D) and pathogen exploitation (Fig. 3C) scenarios, commensal susceptibility improves resistance management (bystander selection) and treatment outcomes (polymicrobial infections) with respect to pathogen density, due to the keystone effect of the commensal supporting pathogen growth (45). In contrast, the competition and commensal exploitation scenarios lead to improved outcomes when commensals are more resistant (Fig. 3A and B). Further exploration (varying the interaction coefficients, see Text S1 and Fig. S5) supports the conjectures above: the qualitative (sign) impact of the commensal susceptibility on pathogen proliferation is dependent on the pathogen incoming interaction parameter (*α_pc_*), while both *α_pc_* and *α_cp_* define the magnitude of the impact (Fig. 4). Specifically, beneficial commensal resistance is maximized for competitive interactions (Fig. 3A and 4), and beneficial commensal susceptibility is maximized for mutualistic interactions (Fig. 3D and 4).

10.1128/mbio.01349-22.9 FIG S5 Ecological outcomes for different ecological relationships between the pathogen and commensal. Colored regions represent the stable steady state (ecological outcomes as described by equations S2 to 4) given corresponding interaction coefficients, *α_pc_* and *α_cp_* and different levels of commensal relative susceptibility (*f*) at high antibiotic exposure (*A* = 1). (A) The commensal is more resistant than the pathogen (*f* = 0.5). Panel B provides a baseline, where either the commensal and pathogen are equally susceptible to antibiotic exposure (*f* = 1) or there is no antibiotic exposure (*A* = 0). (C) The commensal is more susceptible than the pathogen (*f* = 2). Dashed lines represent *α_pc_* = 0 and *α_cp_* = 0. Specific parameters: *r_p_* = 0.5, *r_c_* = 0.5, *k_p_* = 1, *k_c_* = 1, and *x* = 0.1. Download FIG S5, EPS file, 1.2 MB. Copyright © 2022 Wollein Waldetoft et al. 2022 Wollein Waldetoft et al. https://creativecommons.org/licenses/by/4.0/ This content is distributed under the terms of the Creative Commons Attribution 4.0 International license.

### Temporal dynamics, explicit resources, and spatial extension.

**FIG 5:**
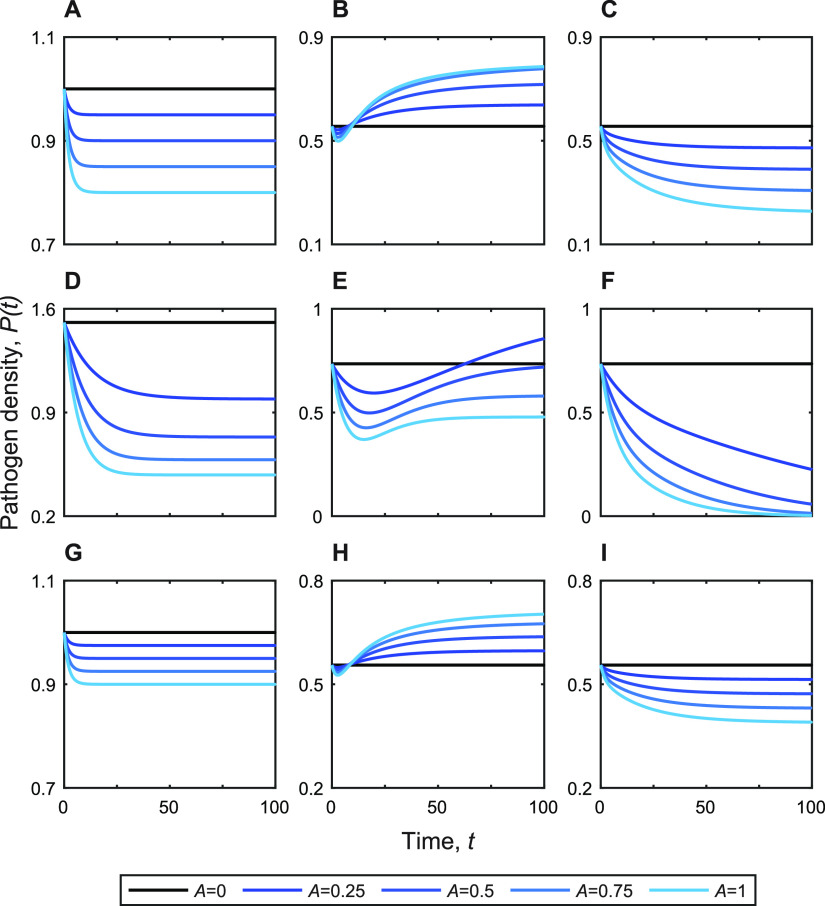
Beneficial resistance is robust to model assumptions. Numerical simulations showing temporal dynamics under a range of antibiotic exposures *A*, denoted by line color. (A to C) Temporal dynamics of the baseline Lotka-Volterra competition model (Fig. 3A, equation 3). (D to F) Temporal dynamics of resource-explicit competition model (46) (Text S1, equation S11). (G to I) Temporal dynamics of spatially extended Lotka-Volterra competition model with a spatial gradient of antibiotic exposure (Text S1, equation S12). (A, D, and G) Pathogen clonal infection. (B, E, and H) Pathogen population plus a drug-susceptible commensal population (high *f*, *f* = 2). (C, F, and I) Pathogen population plus a drug-resistant commensal population (low *f*, *f* = 0.5). The models and parameters used for numerical simulation are defined in Materials and Methods, Fig. 3, Text S1, and Table S1.

Our analyses described above use the standard microbiome modeling convention of a Lotka-Volterra framework, in order to produce mathematically grounded insights into how species interactions shape the costs and benefits of commensal resistance. In this final section, we return to the competition scenario (Fig. 3A) and relax key assumptions to examine the robustness of our conclusions to model and parameter assumptions (Fig. 5).

In Fig. 3A, we assume that pathogen-commensal dynamics have reached the two-species ecological equilibrium. In Fig. 5A to C, we track temporal dynamics on the approach to equilibrium to illustrate our baseline model results on a temporal scale. In Fig. 5A, we track the behavior of the pathogen alone [C(*t*) = 0 for all *t*] under varying antibiotic exposure, following an initial condition of the pathogen equilibrated to the no-antibiotic steady state [*P*(0) = *k_p_*]. In this illustration, we assume the pathogen is partially resistant, so that maximal antibiotic exposure leads to a 20% reduction in pathogen density. In Fig. 5B, we illustrate the competitive release scenario (drug-susceptible commensals) resulting in an expansion of pathogen density with increasing antibiotic (note the change in the *y* axis scale). In Fig. 5C, we illustrate a beneficial resistance scenario (drug-resistant commensals), resulting in more substantial reductions in pathogen density with increasing antibiotic.

In Fig. 5D to F, we turn to a previously published resource-explicit competition model, with experimentally derived parameters (46) (Text S1). Here, we modify the basic model from Hesseler et al. (equation 1 in reference 46) such that we track the dynamics of two competitor populations and one nutrient concentration, setting the internally produced metabolite from their framework to the coexistence equilibrium (see Text S1 and Table S1 for further model and parameter definitions). While this model is more complex and structurally distinct than our baseline framework, we can repeat the same numerical simulations to contrast behaviors across the two models and find support for our basic qualitative conclusions—increasing resistance in commensals increases the suppression of drug-resistant pathogens under antibiotic exposure (Fig. 5E and F).

Both the baseline (Fig. 3, Fig. 4, Fig. 5A to C) and resource-explicit (46) (Fig. 5D to F) models make the assumption that the interacting species are well mixed. In a final step, we extend our baseline model to incorporate spatial extension as a two-dimensional reaction-diffusion process, with a spatial gradient in antibiotic exposure (for partial differential equations, see Text S1). Figure 5G to I show that our qualitative predictions on the benefits of commensal resistance are robust to the incorporation of spatial extension (reported pathogen densities are averaged across space).

## DISCUSSION

Our analysis demonstrates how ecological parameters (growth rates, carrying capacities, interaction coefficients) combine to determine the potential benefits of commensal resistance and the risk of competitive release. In the context of competitive interactions (Fig. 3A), we show that the benefits of commensal resistance (low *f*) are 3-fold: (i) a reduced likelihood of competitive release (equation 1); (ii) a reduced pathogen density given continued coexistence equilibrium (equation 2); and (iii) a reduced likelihood of pathogen dominance and elimination of the commensal population (red regions in Fig. 3A and Fig. S3 and S4). Given certain ecological parameters, these benefits can prevent a previously rare or invading pathogen from colonizing or can help to eliminate an already established pathogen (Fig. S1 and S2).

10.1128/mbio.01349-22.7 FIG S3 The pathogen has a higher growth rate than the commensal, *r_p_* > *r_c_*. Contour maps of pathogen equilibrium density, P*, at stable coexistence (equation S7). Red regions in panels A and C indicate where coexistence is unstable and pathogen dominance (equation S2) is stable. Competitive release will occur whenever there is stable coexistence for the chosen ranges of *f* and *A* (*α_pc_* > 0, competitive release threshold: *f* > 0.417) in panels A and B. Color scales are standardized for panels A to C with contour increments of 0.03. Panel D has its own color scale and contour increments of 0.06 to account for larger densities. Specific parameters: *r_p_* = 0.75, *r_c_* = 0.25, *k_p_* = 1, *k_c_* = 1, and *x* = 0.1. Interaction coefficients (*α_pc_*, *α_cp_*) for panels A to D are consistent with Fig. 3 and Table S3. Download FIG S3, EPS file, 2.1 MB. Copyright © 2022 Wollein Waldetoft et al. 2022 Wollein Waldetoft et al. https://creativecommons.org/licenses/by/4.0/ This content is distributed under the terms of the Creative Commons Attribution 4.0 International license.

10.1128/mbio.01349-22.8 FIG S4 The pathogen has higher carrying capacity than the commensal, *k_p_* > *k_c_*. Contour maps of pathogen equilibrium density, P*, at stable coexistence (equation S7). Red regions in panels A and C indicate that coexistence is unstable for all *f*, *A* and pathogen dominance (equation S2) is stable. Competitive release will not occur in any interaction scenario for the chosen ranges of *f* and *A*. Color scales are standardized for panels A to C with contour increments of 0.03. Panel D has its own color scale and contour increments of 0.06 to account for larger densities. Specific parameters: *r_p_* = 0.5, *r_c_* = 0.5, *k_p_* = 1.5, *k_c_* = 0.5, and *x* = 0.1. Interaction coefficients (*α_pc_*, *α_cp_*) for panels A to D are consistent with Fig. 3 and Table S3. Download FIG S4, EPS file, 1.7 MB. Copyright © 2022 Wollein Waldetoft et al. 2022 Wollein Waldetoft et al. https://creativecommons.org/licenses/by/4.0/ This content is distributed under the terms of the Creative Commons Attribution 4.0 International license.

We also show that the benefits of commensal resistance are not limited to competitive interactions. Scenarios where commensals inhibit pathogens but are in turn enhanced by pathogen presence (commensal predation/exploitation of pathogens, Fig. 3B) can also provide the benefits (items i to iii) described above, although the magnitude of effects are weakened because of the negative feedback on commensal growth as the pathogen is eliminated (Fig. 4).

In cases where the commensal enhances pathogen growth (Fig. 3C and D), we see opposite effects. Commensal resistance now becomes a liability, as it can drive increases in pathogen density via continued facilitation of pathogen growth. Commensal resistance will still reduce the likelihood of pathogen dominance by preserving the commensal population (benefit iii above, red regions in Fig. 3C and Fig. S3 and S4), yet care has to be taken in assessing whether this remains a benefit. In a two-species community, the commensal in this case can be viewed as a cryptic pathogen due to its pathogen-facilitating behavior (47), making its eradication a beneficial outcome. In a more complex community where the commensal interacts with other species as well—possibly inhibiting additional rare or opportunistic pathogens—this becomes a key consideration in optimizing resistance.

By identifying potential benefits of resistance, our analysis provides support for transforming resistance management from a minimization problem to an optimization problem, requiring a balancing of benefits against the established costs of resistance (Fig. 1). To provide a basic illustration of how to approach an optimization problem mathematically, we consider a bystander exposure scenario. The exposed microbiome contains a pathogen, which is asymptomatically carried, but associated with a risk of later infection, and a commensal, which being a commensal has a smaller risk of infection. We take the competition scenario presented in Fig. 3A and attach risk weightings to the equilibrium abundance of pathogens P* (per-capita weighting *w_p_*) and commensals C*(per-capita weighting *w_c_*). In the absence of horizontal gene transfer (HGT), given that pathogens are more virulent (*w_p_α_pc_* > *w_c_*), we find that the balance of costs and benefits tends to favor sufficient commensal resistance to drive the pathogen extinct when the effect of the commensal on the pathogen is inhibitory (Fig. S6A). Adding an additional risk due to HGT of resistance genes can produce a peak of total infection risk at intermediate commensal resistance (maximizing the risk of HGT by preserving both commensals and pathogens) but also preserves the result of minimal risk given sufficient commensal resistance to exclude the pathogen (Fig. S6B). We note that this analysis is a baseline case and that additional complexities (e.g., per-capita weightings depending on the strength of commensal resistance) can change the specific results (see Text S1).

10.1128/mbio.01349-22.10 FIG S6 Balancing costs and benefits of commensal resistance via impacts on commensal and pathogen infection risk. Plots of organism-specific infection risk (dashed lines) and total infection risk (solid lines) as a function of commensal relative susceptibility to antibiotics, *f*. (A) Pathogen infection risk [red, w_p_P*(*f*)] and commensal infection risk [blue, w_c_C*(*f*)], given no HGT. (B) Including HGT introduces another risk class, due to infection by the pathogen with additional HGT-acquired resistance [purple, w_h_βP*(*f*)C*(*f*)]. The pathogen infection risk (red dashed line) captures the benefit of increasing commensal resistance (decreasing *f* → decreasing risk). The commensal infection risk (blue dashed line) captures the non-HGT cost of increasing commensal resistance (decreasing *f* → increasing risk). The purple dashed line represents the infection risk associated with the HGT resistant pathogen where *w_h_β* combines the rate of HGT (*β*) and the risk of HGT resistant pathogen infection (*w_h_*). The solid black line is the total infection risk for the community, defined by summing the contribution from each population (equation S13). For additional model details, see Text S1. Specific parameters: *r_p_*, *r_c_*, *k_p_*, *k_c_*, *α_pc_*, *α_cp_*, and *x* are as defined in Fig. 3A; *A* = 1, *w_p_* = 1, *w_c_* = 0.1, *w_h_β* = 5. Download FIG S6, EPS file, 0.7 MB. Copyright © 2022 Wollein Waldetoft et al. 2022 Wollein Waldetoft et al. https://creativecommons.org/licenses/by/4.0/ This content is distributed under the terms of the Creative Commons Attribution 4.0 International license.

In the following sections, we discuss applied contexts of our beneficial resistance perspective, focusing first on resistance management in the context of bystander selection (following incidental antibiotic exposures in nontarget body sites [30]).

### Optimal spectrum of resistance.

Our mathematical model focuses on relative resistances to a single antibiotic treatment. Yet resistance mechanisms also vary in the spectrum of antibiotics against which they protect. So, for example, different bacterial beta-lactamase enzymes confer resistance to different subsets of the beta-lactam family of antibiotics. What does our logic say about the optimal spectrum of resistance? Consider a bystander selection scenario, where an individual undergoes antibiotic treatment with amoxicillin for an acute upper respiratory tract infection (URTI). In addition to clearing the target URTI, this treatment will also expose a resident gut community of E. coli strains to amoxicillin. Consider in a first case that this community consists of an amoxicillin-susceptible commensal E. coli strain (*C* in Fig. 2, equation 3) and an opportunistically pathogenic E. coli strain (*P*), expressing an extended-spectrum beta-lactamase (ESBL). By inhibiting the sensitive strain, exposure to amoxicillin risks inducing competitive release of the ESBL strain (48). If this occurs, this amoxicillin exposure has increased the abundance of a strain that is resistant not only to amoxicillin, but to 3^rd^-generation cephalosporins as well (49).

Consider now a parallel scenario, where the commensal strain is no longer fully sensitive but has narrow-spectrum resistance to amoxicillin. Now, the amoxicillin exposure will not lead to competitive release of the ESBL pathogen strain (lower *f* in Fig. 3A), limiting both the within-patient and epidemiological spread of ESBL E. coli. This thought experiment suggests that for countries where aminopenicillins are widely prescribed for common conditions, optimal commensal resistance may involve narrow-spectrum aminopenicillin resistance.

### Optimal degree of resistance.

The examples described above consider a binary state of resistance or susceptibility to amoxicillin in the commensal strain. However, resistance is a quantitative trait, and the degree of resistance (e.g., MIC) required to prevent competitive release depends on the concentration of the antibiotic to which the bacteria are exposed. This, in turn, varies across anatomical sites (33). If the antibiotic concentration at a commensal site is substantially lower than at typical infection sites, there may be a degree of resistance that is just right, such that it allows a commensal strain to endure antibiotic exposure at the commensal site and, thus, prevents competitive release of more resistant or pathogenic strains or species, but still leaves the commensal susceptible to the higher concentration of drug attained at infection sites, should it cause opportunistic infection. For bacteria that mostly reside as commensals in the gut but occasionally cause urinary tract infection, this scenario seems quite plausible given that many antibiotics have renal clearance.

### Optimal mechanism and context of resistance.

As outlined in the introductory section and Fig. 1, a problem with resistance in commensals is that resistance genes can be horizontally transferred to pathogens. (A recent experimental evolution study calls into question the magnitude of this risk, showing that while E. coli expanded and evolved resistance in sterilized human microbiome environments, E. coli growth was suppressed and resistance evolution was halted in live microbiome environments [21].) Additional problems include the risk that commensals themselves cause opportunistic infections and that a resistance that is optimal in spectrum or degree (see above) evolves to be broader or more effective. Together, these points suggest that the optimal resistance mechanism is one that is not readily transferable to other taxa, is poorly evolvable, and occurs in bacteria with low pathogenic potential.

### Optimal antibiotic spectrum and dosing.

While the resistance of commensals may be leveraged to limit the growth of pathogens in polymicrobial contexts (chronic infections, microbiomes), different means to similar ends would be to refine antibiotic selection and dosing to tune the effect on commensals or to develop novel antibiotics with optimal spectra of activity. These routes are conceptually related to the matter of commensal resistance since both the resistance pattern of the microbiota for a given drug and the effective spectrum of that drug for that microbiota can be construed as the set of potencies (e.g., MICs) of the drug for the microbes in question and are thus two sides of the same coin. This equivalency suggests on first reflection that we can simply focus on narrow-spectrum treatments (e.g., phage therapy) to maximize commensal resistance. We urge caution in the interpretation of this equivalency, as narrow-spectrum treatment alone does not guarantee that appropriate commensal competitors are present.

Antimicrobial treatment not only interacts with the resistance pattern of the microbiota, but also changes that pattern by selecting for resistance. This is the problem of bystander selection (30). In principle, this side effect of treatment (selection for off-target resistance) could have benefits if selection for increased resistance occurs primarily in commensals that compete with potential pathogens. While our analysis suggests that bystander selection is not always and unequivocally damaging, we do not conclude from this that bystander selection should be encouraged, due to obvious risks of increased resistance in potentially damaging opportunistic pathogens (30).

Regarding the tuning of doses, there is a balance to strike between limiting indirect competitive release effects in bystander microbiome sites and ensuring adequate treatment of the target pathogen. Concerning adequate dosing of target acute infections, we refer to the separate debate on under-/overdosing of acute infections in the context of treatment efficacy and resistance management (50–52).

### Additional considerations in a polymicrobial infection treatment context.

For people with chronic polymicrobial infections, we can also ask how our results inform treatment options given a complex community ecological infection setting. The potential for beneficial resistance opens a path toward novel therapeutic strategies that combine defined antibiotics with probiotic adjuvants featuring rationally designed resistances. Table 1 highlights key levers to consider in the design of an optimized probiotic adjuvant paired with a specific antibiotic therapy.

**TABLE 1:** Optimizing the costs/benefits of commensal resistance: potential levers in the design of a probiotic adjuvant to antibiotic treatment

Optimization components	Effect of commensal resistance	Potential levers
Factors to maximize (benefits)	Inhibition of pathogen by commensal	Enhanced resource competition from commensal (22) Enhanced production of pathogen-specific antimicrobials by commensal (5) Enhanced exploitative activity (e.g., predatory) by commensal (60)^*a*^ Limited drug spectrum and dosing to retain commensal resistance in competitors
Factors to minimize (costs)	HGT from commensal to pathogen	Commensal with resistance mechanism with minimal potential for HGT, e.g., structural resistance mechanisms (10)
	Opportunistic infection by commensal	Commensal with low infection potential (dependent on context of specific patient vulnerabilities) Commensal with resistance mechanism with narrow activity (i.e., the strain can be “recalled” by alternate antibiotic treatments, or higher dosing, if necessary) (61)

a We note that commensal exploitation of the pathogen produces weaker benefits of resistance than commensal-pathogen competition (Fig. 4).

### Caveats and extensions.

While our simple mathematical model supports the concept of beneficial resistance in the case of pairwise interactions, it is possible that in more complex communities and environments, the pairwise benefits of resistance could be attenuated or even reversed via other more complex indirect effects (e.g., where species A impacts species B via impacts on a third species, C [53]). It therefore remains an important challenge to assess the positive and negative consequences of resistance in more complex multispecies contexts. Moving toward larger community structures also flags a critical empirical challenge for this emerging research theme—which species (or strain) has what resistance? At present, the study of microbiome resistances (the “resistome”) typically results in a catalogue of resistance determinants with weaker resolution of exactly what species or strain carries what resistance. Recent methodological developments (54) point to novel solutions to this technical challenge.

Our results provide predictions that are directly amenable to experimental testing, most clearly outlined (for the competitor case) in the temporal dynamics Fig. 5. Our model presents the prediction that inserting a resistance gene in the commensal (moving from Fig. 5B to 5C) will qualitatively shift pathogen dynamics from expansion to contraction following antibiotic exposure. We note that, in addition, our model provides distinct qualitative and quantitative predictions given exploitative or mutualistic species interactions (Fig. 3 and 4). Our predictions are also testable on the level of longitudinal patient microbiome studies. For example, given competitive interactions, we expect to see competitive release less often than in cases where commensals are sufficiently resistant to treatment.

### Summary.

Antibiotic use has the side effect that it promotes resistant strains. We suggest that rather than accepting this as a largely unavoidable problem (alternative therapeutic strategies do exist [55–58]), we can leverage resistance enrichment to achieve ends that are more desirable, or at least less undesirable. Our mathematical model generates precise definitions of beneficial commensal resistance in terms of basic ecological and demographic parameters and identifies that beneficial resistance can be maximized when commensals and pathogens compete. More broadly, we argue that mapping the pros and cons of resistances in the microbiome can inform antibiotic risk assessment and resistance management and, ultimately, improve treatment outcomes on both the patient and public health levels (57). We outline key resistance optimization dimensions, including optimization of resistance magnitude, spectrum, and mechanism. While the problems caused by antibiotic-resistant bacteria should be minimized, we argue that the best means to this end may not be to minimize all antibiotic resistance but, rather, to find the optimal compromise between positive and negative aspects of antibiotic resistance.

## MATERIALS AND METHODS

### Model definition.

We utilize a two-species (or strain) Lotka-Volterra competition model of pathogen absolute density *P*(*t*) and a commensal absolute density *C*(*t*) depicted by the schematic in Fig. 2 and defined as

(3a) $$\frac{\mathrm{dP}}{\mathrm{dt}}=r_{p}P\left(1\;-\;\frac{P\;+\;\alpha_{\mathrm{pc}}C}{k_{p}}\right)\;-\;\mathrm{xAP}$$

(3b) $$\frac{\mathrm{dC}}{\mathrm{dt}}=r_{c}C\left(1\;-\;\frac{C\;+\;\alpha_{\mathrm{cp}}P}{k_{c}}\right)\;-\;\mathrm{xf}AC$$

The parameters *α_pc_* and *α_cp_* are limited to the range |*α_ij_*| < 1, allowing both inhibitory (*α_ij_* > 0) and faciliatory (*α_ij_* < 0) interactions but disallowing the strong inhibitory impact of interference competition (*α_ij_* > 1) to avoid complexities of bistable ecological equilibria. All other parameters are assumed to be greater than or equal to zero. Carrying capacities are rescaled to a unit density for a default pathogen in the no-antibiotic case (and restricted to a range of 0 to 2, Table S1) for simplicity (59). Due to our derivation of dimensionless quantities governing model behavior (equations 1 and 2), our analyses are robust to changes in parameterization. Specific parameters were chosen to best graphically illustrate how general relationships between demographic and ecological parameters govern the potential benefits of resistance. Table S1 provides a definition of parameters and a summary of specific parameter values used to generate the figures.

To represent the effects of antibiotic treatment in the model, the pathogen loss rate, *xA*, (equation 3a) increases from 0 to its maximal clearance rate *x* as antibiotic exposure *A* increases from 0 (no antibiotic) to 1 (maximum antibiotic). We defined the commensal loss rate relative to the pathogen, using commensal relative susceptibility *f* (equation 3b), meaning that the commensal is less susceptible to antibiotic effects than the pathogen when *f* < 1, more susceptible when *f* > 1, and equally susceptible when *f* = 1.

Additional information on reference and alternative models is presented in Text S1 and Tables S1 to S3, including the resource-explicit model (equation S11) and the spatially extended model (equation S12).

### Data availability.

The code for analysis was created using MATLAB R2021b–academic use. Figures were produced using MATLAB R2021b–academic use and Adobe Illustrator 2021. Specific parameters are defined in Text S1 and Table S1 and in the figure legends for Fig. 3 and 4 and Fig. S1 to S6. The code is publicly available on GitHub at https://github.com/GaTechBrownLab/commensal_resistance.
