# Supplementary material for: Defining the Benefits of Antibiotic Resistance in Commensals and the Scope for Resistance Optimization
Source: mBio. 2022 Dec 7;14(1):e01349-22. doi: 10.1128/mbio.01349-22 (PMC9972992; doi:10.1128/mbio.01349-22)
Supplement: TABLE S1 [file mbio.01349-22-st001.docx]

**Table S1. Definition of model parameters and summary of specific parameter value ranges used to generate figures.**

| **Parameter** | **Biological Definition** | **Lotka-Volterra_a_** | **Resource Explicit_b_** | **Spatial Extension** |
| --- | --- | --- | --- | --- |
| $r_{p}$ | Pathogen maximal growth rate | $(0, 1]$ | -- | $0.5$ |
| $r_{c}$ | Commensal maximal growth rate | $(0, 1]$ | -- | $0.5$ |
| $k_{p}$ | Pathogen carrying capacity | $(0, 2]$ | -- | $1$ |
| $k_{c}$ | Commensal carrying capacity | $(0, 2]$ | -- | $1$ |
| $\alpha_{pc}$ | Per-capita inhibitory effect of the commensal on the pathogen | $(-1, 1)$ | -- | $0.8$ |
| $\alpha_{cp}$ | Per-capita inhibitory effect of the pathogen on the commensal | $(-1, 1)$ | -- | $0.8$ |
| $x$ | Pathogen maximal clearance rate | $(0, 1]$ | $0.1$ | $0.1$ |
| $f$ | Commensal relative susceptibility | $[0.5, 2.1]$ | $0.5, 2$ | $0.5, 2$ |
| $A$ | Antibiotic exposure | $[0, 1]$ | $[0,1]$ | $[0,1]$ |
| $m_{p}$ | Pathogen maximal growth rate for nutrient $S$ | -- | $0.75$ | -- |
| $k_{p}$ | Pathogen maximal growth rate for additional metabolite $R$ | -- | $0.18$ | -- |
| $m_{c}$ | Commensal maximal growth rate for nutrient $S$ | -- | $0.89$ | -- |
| $a_{p}$ | Pathogen Monod constant for uptake of nutrient $S$ | -- | $0.2$ | -- |
| $l_{p}$ | Pathogen Monod constant for uptake of additional metabolite $R$ | -- | $0.45$ | -- |
| $a_{c}$ | Commensal Monod constant for uptake of nutrient $S$ | -- | $0.22$ | -- |
| $\gamma_{p}$ | Pathogen yield coefficient for $S$ | -- | $1.5$ | -- |
| $\gamma_{c}$ | Commensal yield coefficient for $S$ | -- | $1.53$ | -- |
| $S^{0}$ | Nutrient input concentration | -- | $1$ | -- |
| $D$ | Dilution rate | -- | $0.05$ | -- |
| $D_{p}$ | Pathogen diffusion constant | -- | -- | $0.01$ |
| $D_{c}$ | Commensal diffusion constant | -- | -- | $0.01$ |

_a_Value ranges utilized for analysis of the modified Lotka-Volterra model (Equation 3). We assessed results within these ranges and define specific parameterizations for figures in the corresponding captions.

_b_Parameter values are the experimentally derived values from [6].
