# Supplementary material for: Defining the Benefits of Antibiotic Resistance in Commensals and the Scope for Resistance Optimization
Source: mBio. 2022 Dec 7;14(1):e01349-22. doi: 10.1128/mbio.01349-22 (PMC9972992; doi:10.1128/mbio.01349-22)
Supplement: TEXT S1 [file mbio.01349-22-s0001.pdf]

Text S1. Supplemental Information

Following from the Materials and Methods, we describe the mathematical analysis of the ordinary differential equation model shown in Figure 2 and defined by Equations 3a and 3b:

$$\frac{dP}{dt} = r_p P(1 - \frac{P + \alpha_{pc}C}{k_p}) - xAP, \tag{3a}$$

$$\frac{dC}{dt} = r_c C(1 - \frac{C + \alpha_{cp}P}{k_c}) - xfAC. \tag{3b}$$

1. Qualitative Analysis

1.1. Antibiotic exposure

We analyze the qualitative behavior of Equation 3, assuming antibiotic exposure $A > 0$. We describe the qualitative behavior by defining equilibrium points or steady states as the pathogen and commensal densities that satisfy $\frac{dP}{dt} = 0$ and $\frac{dC}{dt} = 0$ (where the species densities are not changing in time). We denote the pathogen and commensal densities at equilibrium as (P^*, C^*). Here, we have the four qualitative steady states or equilibria, defined in terms of ecological outcomes:

- **joint extinction**:

$$(0, 0) \tag{S1}$$

- **pathogen dominance**:

$$\Big(\frac{k_p(r_p - xA)}{r_p}, 0\Big) \tag{S2}$$

- **commensal dominance**:

$$\Big(0, \frac{k_c(r_c - xfA)}{r_c}\Big) \tag{S3}$$

- **co-existence**:

$$\Big(\frac{r_c k_p(r_p - xA) - \alpha_{pc} r_p k_c(r_c - xfA)}{r_p r_c(1 - \alpha_{pc}\alpha_{cp})}, \frac{r_p k_c(r_c - xfA) - \alpha_{cp} r_c k_p(r_p - xA)}{r_p r_c(1 - \alpha_{pc}\alpha_{cp})}\Big) \tag{S4}$$

In addition to completing stability analysis, we ensure that the pathogen and commensal densities

are biologically relevant, meaning that the equilibrium points are non-negative and each state is distinct. In the case of joint extinction, both species will have zero population density at equilibrium ($P^* = 0$, $C^* = 0$). Given single-species dominance, one species will have a positive population and the other will be zero ($P^* = 0$, $C^* > 0$ or $P^* > 0$, $C^* = 0$), and co-existence will be defined as both species having a positive population density ($P^* > 0$, $C^* > 0$). Thus, the joint extinction state is always biologically relevant and the single-species dominance equilibria will be relevant when the persisting species has a maximal growth rate greater than loss due to antibiotic exposure ($r_p > xA$ for pathogen dominance and $r_c > xfA$ for commensal dominance).

Given our definition for biological relevance at co-existence equilibrium, $P^* > 0$ and $C^* > 0$, we define the following conditions:

For positive pathogen density at the co-existence equilibrium (Equation S4), we have:

$$\begin{aligned} P^* = \frac{r_c k_p (r_p - xA) - \alpha_{pc} r_p k_c (r_c - xfA)}{r_p r_c (1 - \alpha_{pc}\alpha_{cp})} &> 0 \\ r_c k_p (r_p - xA) - \alpha_{pc} r_p k_c (r_c - xfA) &> 0 \\ r_c k_p (r_p - xA) &> \alpha_{pc} r_p k_c (r_c - xfA). \end{aligned}$$

Given that α_{pc} can be positive, negative, or zero, we first consider $\alpha_{pc} > 0$,

$$\frac{r_c k_p}{\alpha_{pc} r_p k_c}(r_p - xA) - r_c > -xfA$$

and switching the orientation of the inequality, this simplifies to

$$f > \frac{r_c}{xA} - \frac{r_c k_p}{\alpha_{pc} r_p k_c xA}(r_p - xA) = \frac{\alpha_{pc} r_p r_c k_c - r_c k_p (r_p - xA)}{\alpha_{pc} r_p k_c xA}.$$

In the case where $\alpha_{pc} < 0$, the sign will be flipped, giving $f < \frac{\alpha_{pc} r_p r_c k_c - r_c k_p (r_p - xA)}{\alpha_{pc} r_p k_c xA}$. In the case

where $\alpha_{pc} = 0$, the condition on P^* will simplify to

$$P^* = \frac{r_c k_p (r_p - xA)}{r_p r_c} > 0$$
$$r_p - xA > 0$$
$$r_p > xA.$$

For positive commensal density at the co-existence equilibrium (Equation S4), we have:

$$C^* = \frac{r_p k_c (r_c - xfA) - \alpha_{cp} r_c k_p (r_p - xA)}{r_p r_c (1 - \alpha_{pc}\alpha_{cp})} > 0$$
$$r_p k_c (r_c - xfA) > \alpha_{cp} r_c k_p (r_p - xA)$$
$$-xfA > \frac{\alpha_{cp} r_c k_p}{r_p k_c}(r_p - xA) - r_c$$
$$f < \frac{r_c}{xA} - \frac{\alpha_{cp} r_c k_p}{r_p k_c xA}(r_p - xA) = \frac{r_p r_c k_c - \alpha_{cp} r_c k_p (r_p - xA)}{r_p k_c xA}.$$

As there is no division by α_{cp} (such as there is in the above pathogen density case with α_{pc}), the condition on f will be the same regardless of the sign of α_{cp}.

In completing our qualitative analysis of the model, we must satisfy both the constraints defined above for biological relevance, as well as any stability constraints in order for our results to be clinically relevant descriptions of infection communities and their predicted behavior.

To assess the stability of each steady state, we define the Jacobian matrix corresponding to Equation 3,

$$J(P^*, C^*) = \begin{bmatrix} r_p(1 - \frac{P^* + \alpha_{pc} C^*}{k_p}) - \frac{r_p}{k_p} P^* - xA & -\frac{\alpha_{pc} r_p}{k_p} P^* \\ -\frac{\alpha_{cp} r_c}{k_c} C^* & r_c(1 - \frac{C^* + \alpha_{cp} P^*}{k_c}) - \frac{r_c}{k_c} C^* - xfA \end{bmatrix}. \quad \text{(S5)}$$

We linearize the matrix (Equation S5) at each equilibrium and use the conditions: $\mathrm{tr}(J) < 0$ and $\det(J) > 0$, or the eigenvalues of the matrix: $\lambda_1, \lambda_2 < 0$ (where computationally efficient) to

determine where each state is asymptotically stable. In the context of our model, we interpret stability to mean that the two species community is at or will progress over time towards an ecological outcome (joint extinction, single-species dominance, or co-existence) given any initial density and remain there unless sufficiently perturbed (i.e. by antibiotic treatment) to induce a state transition to another ecological state.

Given we initially assume stable co-existence of the commensal and pathogen (either with population densities at or tending towards equilibrium), we focus on the co-existence equilibrium (Equation S4). As the eigenvalues of Equation S5 evaluated at the co-existence equilibrium are not easily simplifed, we instead utilize the conditions: $\text{tr}(J) < 0$ and $\det(J) > 0$ to define the region of stability. We recall our assumptions, $|\alpha_{pc}| < 1$ and $|\alpha_{cp}| < 1$, which imply $\alpha_{pc}\alpha_{cp} < 1$.

Turning to the stability conditions, we assess $\text{tr}(J) < 0$, first:

$$\frac{r_c k_p(r_p - xA) - \alpha_{pc} r_p k_c(r_c - xfA)}{r_c k_p(\alpha_{pc}\alpha_{cp} - 1)} + \frac{r_p k_c(r_c - xfA) - \alpha_{cp} r_c k_p(r_p - xA)}{r_p k_c(\alpha_{pc}\alpha_{cp} - 1)} < 0.$$

We notice that the denominators of both terms will always be negative for our assumptions ($\alpha_{pc}\alpha_{cp} < 1 \Rightarrow \alpha_{pc}\alpha_{cp} - 1 < 0$) and the numerators of both terms will be always be positive given biological relevance. Thus, the resulting left hand side of the inequality will be less than 0 and the condition, $\text{tr}(J) < 0$, satisfied whenever co-existence is biologically relevant.

We look to satisfy the last stability constraint, $\det(J) > 0$,

$$\frac{-[r_p r_c k_c - \alpha_{cp} r_p r_c k_p + \alpha_{cp} r_c k_p xA - r_p k_c xfA][r_p r_c k_p - \alpha_{pc} r_p r_c k_c - r_c k_p xA + \alpha_{pc} r_p k_c xfA]}{r_p r_c k_p k_c(\alpha_{pc}\alpha_{cp} - 1)} > 0$$

$$[r_p r_c k_c - \alpha_{cp} r_p r_c k_p + \alpha_{cp} r_c k_p xA - r_p k_c xfA][r_p r_c k_p - \alpha_{pc} r_p r_c k_c - r_c k_p xA + \alpha_{pc} r_p k_c xfA] > 0$$

$$\left[r_p k_c(r_c - xfA) - \alpha_{cp} r_c k_p(r_p - xA)\right]\left[r_c k_p(r_p - xA) - \alpha_{pc} r_p k_c(r_c - xfA)\right] > 0.$$

In order for this condition to be satisfied, the expressions in each set of square brackets need to

have the same sign, i.e. both are positive or both are negative,

$$r_p k_c(r_c - xfA) - \alpha_{cp} r_c k_p(r_p - xA) > 0 \text{ and } r_c k_p(r_p - xA) - \alpha_{pc} r_p k_c(r_c - xfA) > 0$$

or

$$r_p k_c(r_c - xfA) - \alpha_{cp} r_c k_p(r_p - xA) < 0 \text{ and } r_c k_p(r_p - xA) - \alpha_{pc} r_p k_c(r_c - xfA) < 0.$$

A closer look reveals that if the expressions in each set of square brackets are positive, they are identical to the conditions satisfied for biological relevance and which ensure $\text{tr}(J) < 0$, whereas the negative case will not result in biologically relevant co-existence. Thus, given our assumptions, the equilibrium $(P^* > 0, C^* > 0)$, will always be stable when biologically relevant.

We complete the same analysis for the remaining three equilibria (Equations S1, S2, S3). The ecological outcomes and the corresponding conditions for stability given antibiotic exposure are summarized in Table S2. For the purpose of our analysis moving forward, we assume an ecological outcome (steady state) is stable if the conditions for both asymptotically stability and biological relevance are met.

1.2. Absence of antibiotic exposure

For the examples of ecological interactions given in the main text, we assume stable co-existence in the absence of antibiotic exposure. Using $A = 0$, Equation 3 simplifies to the Lotka-Volterra competition model without an additional loss term,

$$\frac{dP}{dt} = r_p P(1 - \frac{P + \alpha_{pc} C}{k_p}), \qquad \frac{dC}{dt} = r_c C(1 - \frac{C + \alpha_{cp} P}{k_c}). \tag{S6}$$

It's evident from Equation S6 that commensal relative susceptibility, as characterized by f in Equation 3, does not offer a cost nor benefit to either species in the absence of antibiotics for the analysis in this paper, as we wish to highlight the potential benefit of commensal resistance on a pathogen population in the presence of antibiotics.

2. Quantitative Analysis

2.1. Competitive release

We define competitive release of the pathogen using the gradient, $\partial P^*/\partial A$, as an increase in the pathogen density with respect to an increase in antibiotic concentration ($\frac{\partial P^*}{\partial A} > 0$) at stable co-existence of the pathogen and commensal ($P^* > 0$, $C^* > 0$). Assuming we have stable co-existence, the pathogen density will be

$$P^* = \frac{r_c k_p (r_p - xA) - \alpha_{pc} r_p k_c (r_c - xfA)}{r_p r_c (1 - \alpha_{pc}\alpha_{cp})}. \tag{S7}$$

Taking the partial derivative of Equation S7 with respect to A, we get

$$\frac{\partial P^*}{\partial A} = \frac{x(\alpha_{pc} r_p k_c f - r_c k_p)}{r_p r_c (1 - \alpha_{pc}\alpha_{cp})}. \tag{S8}$$

Thus, we can define the threshold for competitive release as

$$\alpha_{pc} f > \frac{r_c k_p}{r_p k_c}. \tag{S9}$$

We note that this is gives us Equation 1. Simplifying this a step further, we get the conditions for competitive release given the type of effect the commensal has on the pathogen:

- if $\alpha_{pc} > 0$, $f > \frac{r_c k_p}{\alpha_{pc} r_p k_c}$
- if $\alpha_{pc} < 0$, $f < \frac{r_c k_p}{\alpha_{pc} r_p k_c}$
- if $\alpha_{pc} = 0$, $\frac{r_c k_p}{r_p k_c} < 0$

We note that in the second case ($\alpha_{pc} < 0$), the upper bound on f will be negative due to the value of α_{pc}. Given that we assume f to be positive, competitive release isn't possible when $\alpha_{pc} < 0$ (i.e. when the commensal facilitates the pathogen). Biologically, this makes sense. Competitive release implies that another species has an inhibitory effect on the pathogen, and that when the "competitor" is sufficiently eliminated due to antibiotic treatment the pathogen population is able to expand in the competitor's absence. In the case where $\alpha_{pc} = 0$ (no effect of the commensal on the pathogen), all growth parameters are positive, so the condition cannot be satisfied as well.

Thus, our definition supports the biological interpretation that competitive release is only possible when the commensal inhibits the pathogen (i.e. is a competitor).

We also note that when competitive release is possible ($\alpha_{pc} > 0$), differences between the commensal and pathogen species in regards to growth parameters, r and k, will impact the likelihood of competitive release occurring. As the condition for competitive release (Equation S9) is dimensionless (i.e. Equation S9 is a condition on f, a dimensionless scaling parameter), this result is robust to the chosen parameter values because it depends on the relative relationships between parameters versus a specific value. Increasing the pathogen maximal growth rate, r_p, or the commensal carrying capacity, k_c, will lower the bound on f, making competitive release more likely in the chosen parameter space. Increasing the commensal maximal growth, r_c, or the pathogen carrying capacity, k_p, will raise the bound on f, making competitive release less likely. Similarly, changing the magnitude of α_{pc} will make competitive release more likely when α_{pc} is increased or less likely when α_{pc} is decreased.

2.2. Effect of commensal susceptibility on pathogen density

Similarly, we look at how changing commensal susceptibility impacts equilibrium pathogen density at co-existence. Taking the partial derivative of Equation S7 with respect to f, we get

$$\frac{\partial P^*}{\partial f} = \frac{\alpha_{pc} k_c x A}{r_c(1 - \alpha_{pc}\alpha_{cp})}. \tag{S10}$$

We note that setting this equation greater than 0, leads us to Equation 2.

If $\frac{\partial P^*}{\partial f} > 0$, an increase in relative commensal susceptibility leads to an increase in pathogen density at the co-existence equilibrium (or decreasing f leads to decreasing P^*). This means that commensal resistance is beneficial in limiting pathogen density. Given the assumptions that $|\alpha_{ij}| < 1$ and $r_c > 0$, $\frac{\partial P^*}{\partial f} > 0$ simplifies to: $\alpha_{pc} > 0$. Thus, when the commensal inhibits the pathogen, commensal resistance is beneficial in limiting the pathogen burden. In the opposite case ($\alpha_{pc} < 0$, commensal facilitates pathogen), commensal resistance will enhance pathogen density (i.e. decreasing susceptibility leads to increasing pathogen, $\frac{\partial P^*}{\partial f} < 0$). When the commensal has

no effect on pathogen growth, $\alpha_{pc} = 0$, it follows intuitively that commensal resistance will have no impact on pathogen density ($\frac{\partial P^*}{\partial f} = 0$). Again, this is a dimensionless condition (α_{pc} is a dimensionless scaling parameter), showing resistance can be beneficial depending on the sign of the interaction versus given a specific parameter value or magnitude of interaction.

While the existence of a benefit from commensal resistance is governed by the sign of α_{pc}, both interaction coefficients modify the magnitude of the potential benefit of commensal resistance. Figure 4 shows the value of Equation S10 given possible values of α_{pc} and α_{cp}. Here, we see that the magnitude of the impact of commensal resistance is weaker given a mixed sign interaction (i.e. exploitation) versus an equal sign interaction (i.e. competition or mutualism). Mathematically, this is due to the $\frac{1}{(1-\alpha_{pc}\alpha_{cp})}$ term in Equation S10. Having a mixed sign interaction results in $\frac{1}{1-\alpha_{pc}\alpha_{cp}} < 1$, whereas an equal sign interaction gives $\frac{1}{1-\alpha_{pc}\alpha_{cp}} > 1$. Therefore, f will always have a weaker magnitude effect on P^* in the case of a mixed sign interaction versus an equal sign interaction when all parameter magnitudes are consistent. This mathematical definition supports the biological interpretation given in the main text for the magnitude of the benefit of commensal resistance.

The magnitude of the effect of commensal resistance on pathogen density is also positively dependent on commensal carrying capacity (k_c), pathogen maximal clearance rate (x), and antibiotic exposure (A), and inversely impacted by commensal growth rate (r_c), as is seen in Equation S10.

3. Robustness of results

3.1. Variation in growth parameters

In real infection communities, it is unlikely that the commensal and pathogen species will have the same maximal growth rates and carrying capacities (1-4), as we assume for the ecological interaction scenarios in Figure 3 and Table S3. Relaxing these parameters, we find that differences between r_p and r_c, and k_p and k_c alter the bounds for stability (given in Table S2), the threshold for competitive release (as described in Section *2.1*), and the bacterial density at equilibrium (Equations S2-S4). These differences can maximize or minimize the potential benefits of commensal resistance, either

helping to steer towards preferential treatment outcomes or triggering infections that are much harder to control, respectively. We note that the conditions for competitive release and beneficial commensal resistance defined in the main text (Equations 1 and 2) and above (Equations S9 and S10) still hold regardless of the chosen parameterization. Figures S1-S4 show Figure 3 with alternate parameterizations.

Resource competition and commensal exploitation of the pathogen

Commensal resistance remains highly beneficial in controlling the pathogen density and avoiding competitive release. The effect of commensal resistance on limiting pathogen equilibrium density, P^*, at stable co-existence is maximized when the commensal has a growth advantage over the pathogen (comparing panels (A-B) in Figures S1-S4) and can even shift the community from stable co-existence to commensal dominance, eliminating the pathogen population all together, given sufficient resistance or growth advantage in the commensal relative to the pathogen (dark blue regions in panels (A-B) in Figures S1 and S2). If the pathogen has a growth advantage over the commensal (panels (A-B) in Figures S3 and S4), competitive release may be unavoidable regardless of commensal resistance or antibiotic exposure (Figure S3) and is more likely to lead to stable pathogen dominance (red regions in panel (A) in Figures S3 and S4). Similar changes occur given competition between the commensal and pathogen and commensal exploitation of the pathogen; however, the benefits of commensal resistance are not as strong as in the mutually inhibitory case due to the difference in ecological feedback of antibiotic-induced pathogen clearance.

Pathogen exploitation of the commensal

Regardless of changes to r and k, increased antibiotic suppresses pathogen density and pathogen growth is maximized at high commensal resistance. However, because the commensal facilitates the pathogen, pathogen equilibrium density in the pathogen dominant state will be lower than pathogen density at co-existence. Therefore, commensal resistance becomes beneficial in preventing a transition to pathogen dominance and preserving the commensal population when commensal growth is low or when pathogen carrying capacity is higher than commensal carrying capacity ($k_p > k_c$) (red regions in panel (C) in Figures S3 and S4). This "benefit" is somewhat trivial, given that lower pathogen density is always preferable in infection control and the commensal population

indirectly exerts a negative impact on the host, making it a "cryptic pathogen" (5) in this scenario.

Mutualism

Community outcomes given antibiotic treatment are mostly unchanged in the case of a mutualistic interaction (panel (D) in Figures S1-S4). This is largely due to the fact that co-existence is extremely stable and robust given mutually faciliatory inter-specific interaction. The pathogen density at co-existence changes slightly due to which species has a growth advantage over the other, but the trends regarding antibiotic exposure and commensal susceptibility described in the main text are preserved. Like in the pathogen exploitation case, the value of P^* at the pathogen dominant equilibrium point will be less than that at co-existence, meaning commensal resistance will be beneficial in preventing a state transition to pathogen dominance (resulting in elimination of the commensal) at high antibiotic exposure when the pathogen has a sufficiently large growth advantage over the commensal.

Changes in competitive release are fairly straightforward given Equation S9, as described in *2.1.* The result that competitive release can only occur when the effect of the commensal on the pathogen is inhibitory, $\alpha_{pc} > 0$, and co-existence is stable remains unchanged by variation in growth parameters.

3.2. Variation in inter-specific interaction parameters

While we use specific parameterizations to represent different ecological relationships between the commensal and pathogen, we also show that these results are generalizable to the type of ecological interaction in general. Figure S5 depicts the qualitative outcomes of the model given different levels of commensal relative susceptibility f and antibiotic exposure A with respect to α_{pc} (x-axis) and α_{cp} (y-axis). Our analysis assumes exploitative type interactions, represented by $|\alpha_{ij}| < 1$, to favor stable co-existence. Relaxing this assumption, we see that taking larger magnitude values ($\alpha_{ij} > 1$) will allow for a greater likelihood of stable single-species dominance and introduces the possibility of bistability between single-species dominant states.

Generally, for exploitative type interactions, our model shows that stable co-existence is likely to

occur regardless of antibiotic exposure or relative commensal susceptibility. However, for values of α_{pc} and α_{cp} closer to 1, we see that commensal resistance is helpful in preventing stable pathogen dominance (panel (A) of Figure S5, $f = 0.5$, the red region shrinks along the α_{cp}-axis compared to panel (C) of Figure S5, $f = 2$, where it expands).

3.3. Variation of model form

In order to assess the robustness of our results to the model form, we implemented numerical simulations or "experiments" with two additional model forms and compared the results with our qualitative predictions. This was in an effort to ensure that our results are not bound to the assumptions or form of the modified Lotka-Volterra equations (Equation 3).

Parameters for these models can be found in Table S1. The Lotka-Volterra and resource explicit models were simulated using the Euler method with $dt = 0.001$. Initial conditions were defined by calculating the pathogen only and co-existence equilibriums with no antibiotic exposure, either mathematically or via simulation (Figure 5A: $P(0) = 1$, $C(0) = 0$, Figure 5B-C: $P(0) = 0.5556$, $C(0) = 0.5556$, Figure 5D: $P(0) = 0.9867$, $C(0) = 0$, $S(0) = 0.0133$, Figure 5E-F: $P(0) = 0.0897$, $C(0) = 0.8970$, $S(0) = 0.0133$). The spatially extended model was simulated using a two-dimensional Crank-Nicolson scheme on an $L \times L$ grid with periodic boundary conditions and simulation parameters: $L = 100$, $dx = dy = 0.5$, and $dt = 0.1$. The initial conditions were defined similarly as in the ordinary differential equation case (Figure 5G: $P(0) = 1$, $C(0) = 0$ at each grid location, Figure 5H-I: $P(0) = 0.5556$, $C(0) = 0.5556$ at each grid location). Antibiotic exposure A was simulated as a linear spatial gradient along the x-axis. We report pathogen density $P(t)$ as the average in space.

Resource explicit model

Focusing on the resource competition scenario, we implemented a resource explicit model of bacterial growth in a continuous flow environment (chemostat) based off of the model form and experimentally defined parameters of [6]. We make one simplification, setting the dynamics of the internally produced metabolite to equilibrium, in order to eliminate the additional cross-feeding relationship between the competitors while still allowing the competitors to progress to stable co-existence.

In an experimental system, this could be interpreted as supplementing the growth media with an additional metabolite. We define the model, adding a term for antibiotic exposure (such as in Equation 3), as

$$\frac{dP}{dt} = P\left(\frac{m_p S}{a_p + S} + \frac{k_p R}{l_p + R} - dR - D - xA\right) \tag{S11a}$$

$$\frac{dC}{dt} = C\left(\frac{m_c S}{a_c + S} - dR - D - xfA\right) \tag{S11b}$$

$$\frac{dS}{dt} = D(S^0 - S) - \frac{1}{\gamma_p}\frac{m_p S}{a_p + S}P - \frac{1}{\gamma_c}\frac{m_c S}{a_c + S}C \tag{S11c}$$

where $P(t)$ and $C(t)$ are the absolute densities of the pathogen and commensal populations, respectively, $S(t)$ is the concentration of limiting resource—in this case, glucose—, and R is the value of the additional metabolite ($R = 0.0107$). Parameter definitions and values are given in Table S1. This provides a mechanistic and experimentally grounded example of the resource competition scenario of Equation 3 that we discuss in detail in the main text and above. Figure 5D-F shows that the qualitative results of the Lotka-Volterra model (Figure 5A-C)—mainly prevention of competitive release and limited pathogen burden—are preserved with additional model complexity.

If we assess the model including the dynamics of the additional metabolite ($R(t)$), we can address the case of the pathogen species exploiting the commensal species via cross-feeding. In this case, we see the opposite scenario where the commensal facilitates the pathogen and the results in the main text continue to hold qualitatively: commensal resistance is a cost.

Spatially extended model

Another major assumption of the modified Lotka-Volterra model form that we use is spatial homogeneity. For real human microbiomes, this is unlikely to be the case; however, for our purposes, we look to show that by adding spatial dynamics in the form of diffusion and an antibiotic gradient the qualitative results still hold. We acknowledge that additional means of realistic spatial dynamics and spatiotemporal heterogeneity may alter outcomes; however, as we view these models as a simplified model system versus a complete recapitulation of within host polymicrobial dynamics, this is beyond the scope of this work.

To define the spatial dynamics, we utilize a two-dimensional reaction-diffusion model, where the reaction dynamics are defined by Equation 3 and the parameters used in Figures 3A. We also introduce a linear, one-dimensional antibiotic gradient in space that is constant throughout time. The model is defined,

$$\frac{\partial P}{\partial t} = r_p P(1 - \frac{P + \alpha_{pc} C}{k_p}) - xAP + D_p \nabla^2 P \tag{S12a}$$

$$\frac{\partial C}{\partial t} = r_c C(1 - \frac{C + \alpha_{cp} P}{k_c}) - xfAC + D_c \nabla^2 C. \tag{S12b}$$

where $P = P(x, y, t)$, $C = C(x, y, t)$, $A = A(x)$ and $\nabla^2 = (\frac{\partial^2}{\partial x^2} + \frac{\partial^2}{\partial y^2})$ (we note that in these definitions x is a spatial variable). Again, we see that our qualitative results hold (Figure 5G-I). While not pictured, we altered the interaction coefficients (α_{pc} and α_{cp}) for the case of commensal exploitation of the pathogen (parameters from Figure 3B) in the same framework and found that the qualitative results were also preserved, although commensal resistance continued to provide a smaller magnitude effect in this case as compared to the case where the species compete.

4. Optimization problem

While our analysis focuses on identifying the potential benefits of commensal resistance, we acknowledge that there are established costs in having resistant commensals—predominantly infection by the commensal and horizontal gene transfer (HGT) of resistance genes (Figure 1). To illustrate how costs and benefits of commensal resistance can be combined in an explicit optimization framework, we develop a simple optimization model (Figure S6). We note that our results are designed to provide a proof-of-principle that the costs and benefits outlined in Figure 1 can be combined in a single model framework, and we stress that an actionable optimization model would require more detailed attention to a specific infection context to guide appropriate model selection and parameterization.

We outline our approach based on the competition scenario in Figure 3A, where we seek to optimize the commensal relative susceptibility, f. Using the co-existence equilibrium values, P^* and C^* defined in Equation S4, as a function of commensal relative susceptibility f (all other parameter

values defined and fixed), we can represent the benefits and costs of commensal resistance via the relative risks of infection at different levels of f (Figure S6). To define the risk of infection, we introduce per-capita risk weighting coefficients, w_p, w_c, and $w_h\beta$, to scale the equilibrium densities for the pathogen, commensal, and population of HGT resistant pathogens, respectively. We note that for HGT resistant pathogens the weight term includes the risk of infection w_h, as well as the rate of HGT β. We assume that, in general, $w_h\beta > w_p > w_c$, as HGT resistant pathogen infections are most dangerous. In order to find the optimal resistance, we look to minimize the net risk of infection and define the problem as,

$$\min_{f} \quad w_p P^*(f) + w_c C^*(f) + w_h \beta C^*(f) P^*(f) \tag{S.13}$$

where the terms describe the risk of infection due to each population, with HGT captured here as a simple mass action term dependent on the interaction of the commensal and pathogen populations. For biological relevance, we note that $P^*, C^* \geq 0$.

Figure S6 depicts the risks of infection as a function of f given the pathogen-commensal competition scenario (parameters from Figure 3A). In Figure S6A (no HGT), we see that increasing commensal resistance (decreasing f) generates increasing costs (increased risk of commensal infection, blue dashed line) and increasing benefits (decreased risk of pathogen infection, red dashed line) with the net effect (black solid line) resulting in reduced risk with increasing commensal resistance. Adjusting the weights will alter this outcome quantitatively, but the qualitative result will hold unless the risk associated with the commensal exceeds that of the pathogen multiplied by effect of the commensal on the pathogen (low f is optimal when $w_p\alpha_{pc} > w_c$).

In panel (B) of Figure S6, we introduce the potential for resistance HGT and see that the risk of an HGT resistant pathogen infection is maximized at intermediate resistance values due to the density of both populations, but is less when one species is largely excluded. However, low f is still favorable in reducing the net risk in this specific illustration, as the pathogen population tends to extinction, HGT is reduced, and only the minimal risk of commensal infection remains. While this is reasonable biologically—larger commensal and pathogen populations will lead to more

HGT—this representation of HGT is simplistic. The risk of HGT will be present even when the microbiota is not exposed to antibiotics, the risk of infection w_h will likely itself be dependent on f (transmission of greater resistance leads to greater risk, i.e. decreasing f leads to increasing w_h, flipping the result and making higher f optimal given sufficient risk), and HGT has implications for other bugs in the microbiota.

References

1. Vieira-Silva S, Rocha EPC. 2010. The Systemic Imprint of Growth and Its Uses in Ecological (Meta)Genomics. PLoS Genet 6:e1000808.

2. Weissman JL, Hou S, Fuhrman JA. 2021. Estimating maximal microbial growth rates from cultures, metagenomes, and single cells via codon usage patterns. Proc Natl Acad Sci U S A 118:e2016810118.

3. Stein RR, Bucci V, Toussaint NC, Buffie CG, Rätsch G, Pamer EG, Sander C, Xavier JB. 2013. Ecological modeling from time-series inference: insight into dynamics and stability of intestinal microbiota. PLoS Comput Biol 9:e1003388-e1003388.

4. Anderson JD, Eftekhar F, Aird MY, Hammond J. 1979. Role of bacterial growth rates in the epidemiology and pathogenesis of urinary infections in women. J Clin Microbiol 10:766-771.

5. Eswarappa SM, Estrela S, Brown SP. 2012. Within-Host Dynamics of Multi-Species Infections: Facilitation, Competition and Virulence. PLoS One 7:e38730.

6. Hesseler J, Schmidt JK, Reichl U, Flockerzi D. 2006. Coexistence in the chemostat as a result of metabolic by-products. J Math Biol 53:556-584.
