# Supplementary material for: Defining the Benefits of Antibiotic Resistance in Commensals and the Scope for Resistance Optimization
Source: mBio. 2022 Dec 7;14(1):e01349-22. doi: 10.1128/mbio.01349-22 (PMC9972992; doi:10.1128/mbio.01349-22)
Supplement: TABLE S2 [file mbio.01349-22-st002.docx]

**Table S2. Ecological outcomes and stability conditions given antibiotic exposure (Equation 3).**

| **Ecological Outcome** | **Equilibrium Point** | **Stability Conditions** |
| --- | --- | --- |
| Joint extinction | $(0, 0)$ | $r_{c}<xfA$ and $r_{p}<xA$ |
| Pathogen dominance | $\left( \frac{k_{p}\left( r_{p}-xA \right)}{r_{p}}, 0 \right)$ | $r_{p}-xfA>0$ and $f> \frac{r_{p}r_{c}k_{c}-\alpha_{cp}r_{c}k_{p}(r_{p}-xA)}{r_{p}k_{c}xA}$ |
| Commensal dominance | $\left( 0, \frac{k_{c}\left( r_{c}-xfA \right)}{r_{c}} \right)$ | $r_{c}-xfA>0$ and   - If $\alpha_{pc}>0$, $f< \frac{\alpha_{pc}r_{p}r_{c}k_{c}-r_{c}k_{p}(r_{p}-xA)}{\alpha_{pc}r_{p}k_{c}xA}$ or - If $\alpha_{pc}<0$, $f>\frac{\alpha_{pc}r_{p}r_{c}k_{c}-r_{c}k_{p}(r_{p}-xA)}{\alpha_{pc}r_{p}k_{c}xA}$ or - If $\alpha_{pc}=0$, $r_{p}<xA$ |
| Coexistence | $\left( \begin{aligned} \frac{r_{c}k_{p}\left( r_{p}-xA \right)- \alpha_{pc}r_{p}k_{c}\left( r_{c}-xfA \right)}{r_{p}r_{c}\left( 1-\alpha_{pc}\alpha_{cp} \right)}, \\ \frac{r_{p}k_{c}\left( r_{c}-xfA \right)- \alpha_{cp}r_{c}k_{p}\left( r_{p}-xA \right)}{r_{p}r_{c}\left( 1-\alpha_{pc}\alpha_{cp} \right)} \end{aligned} \right)$ | $f< \frac{r_{p}r_{c}k_{c}-\alpha_{cp}r_{c}k_{p}(r_{p}-xA)}{r_{p}k_{c}xA}$, and   - If $\alpha_{pc}>0$, $f>\frac{\alpha_{pc}r_{p}r_{c}k_{c}-r_{c}k_{p}(r_{p}-xA)}{\alpha_{pc}r_{p}k_{c}xA}$ or - If $\alpha_{pc}<0$, $f<\frac{\alpha_{pc}r_{p}r_{c}k_{c}-r_{c}k_{p}(r_{p}-xA)}{\alpha_{pc}r_{p}k_{c}xA}$ or - If $\alpha_{pc}=0$, $r_{p}>xA$ |
