# Supplementary material for: Defining the Benefits of Antibiotic Resistance in Commensals and the Scope for Resistance Optimization
Source: mBio. 2022 Dec 7;14(1):e01349-22. doi: 10.1128/mbio.01349-22 (PMC9972992; doi:10.1128/mbio.01349-22)
Supplement: TABLE S3 [file mbio.01349-22-st003.docx]

**Table S3. Summary of four types of possible ecological interaction scenarios.**

| **Ecological Interaction** | **Inter-specific Interaction Coefficients** $\boldsymbol{(}\boldsymbol{\alpha}_{\boldsymbol{pc}}\boldsymbol{,}\boldsymbol{\alpha}_{\boldsymbol{cp}}\boldsymbol{)}$ | **Condition for Competitive Release** | **Commensal resistance beneficial?** |
| --- | --- | --- | --- |
| Resource competition | $(0.8, 0.8)$ | $f>1.25$ | Yes |
| Commensal exploitation of pathogen | $(0.8, -0.8)$ | $f>1.25$ | Yes |
| Pathogen exploitation of commensal | $(-0.8, 0.8)$ | NA, $\alpha_{pc}<0$ | No |
| Mutualism | $(-0.8, -0.8)$ | NA, $\alpha_{pc}<0$ | No |
